# Isolated Leptomeningeal Metastasis of Breast Cancer During Neo-Adjuvant Chemotherapy: A Case Report

**DOI:** 10.7759/cureus.25555

**Published:** 2022-05-31

**Authors:** Aaliya Uddin, Mohammed Bilal, Philip Idaewor, Akatya Sinha, Abdalla Saad Abdalla Al-Zawi

**Affiliations:** 1 Plastic and Reconstructive Surgery, Morriston Hospital, Swansea, GBR; 2 Emergency Medicine, North Bristol NHS Trust, Bristol, GBR; 3 Cellular Pathology/Histopathology, Basildon and Thurrock University Hospital, Basildon, GBR; 4 General Surgery, Mahatma Gandhi Missions (MGM) Medical College, Mumbai, IND; 5 General and Breast Surgery, Mid and South Essex University Hospital Group, Basildon, GBR; 6 General and Breast Surgery, Basildon and Thurrock University Hospital, Basildon, GBR; 7 General and Breast Surgery, Anglia Ruskin University, Chelmsford, GBR

**Keywords:** invasive ductal breast carcinoma, chemotherapy, brain metastasis, leptomeningeal carcinomatosis, breast cancer

## Abstract

The incidence of symptomatic brain metastasis from breast cancer ranges from ~10% to 15%. Brain parenchymal metastasis comprises most of this and has been studied more extensively, whereas isolated leptomeningeal carcinomatosis (LC) is exceedingly rare. The diagnosis is most commonly made by lumbar puncture and cerebrospinal fluid (CSF) cytology, although it is persistently negative in about 10% of patients, and hence its pre-mortem diagnosis remains difficult and controversial. There are limited therapeutic options available making the prognosis abysmal. It has been reported that locally responsive breast cancers on chemotherapy can develop CNS metastasis; the blood-brain barrier and the unique brain microenvironment are hypothesized to promote distinct molecular features in such CNS metastases. We present a 37-year-old female with a large triple-negative, node-positive grade three invasive ductal carcinomas with Ki-67 70%. Despite the local response to neoadjuvant chemotherapy, she developed rapidly worsening multiple neurological symptoms. MRI brain showed leptomeningeal enhancement and CSF cytology results were negative with inconclusive other CSF studies. The patient deteriorated very rapidly and a post-mortem diagnosis of isolated LC was made. The notable aspects of this case include the development of a rapidly progressive isolated LC despite the good local response to the chemotherapy, which requires further studying. As the currently available diagnostic and therapeutic tools have limitations, research can be critical in providing better outcomes for this fatal disease.

## Introduction

About one-fifth of all cancer patients will develop brain metastatic disease, and the brain metastatic tumors vastly outnumber primary brain neoplasms the most frequent primary sites for brain metastases occurring in patients with lung, colonic, renal, and breast cancers in addition to melanoma [1,2]. The sites in the central nervous system metastasis can include the brain parenchyma, dura, skull bone, spinal cord, peripheral nerves, and the leptomeninges[3]. The Global Cancer Statistic 2020 report from the American Cancer Society revealed that breast cancer is regarded as the most frequent malignancy globally after the exclusion of the non-melanomatous skin cancers, and the WHO's recent reports show that breast cancer occupies the fifth place in the cancer-related mortality list after lung, colon, liver, and gastric cancers [4,5]. The 2017 UK data reports breast malignancy as the most often detected cancer in females (after exclusion of non-melanoma skin cancer), with more than 55,000 new cases diagnosed yearly, this means, that breast cancer accounts for 15% of all newly diagnosed cancers and is also considered to be the most frequently encountered cancer in women [6].

Cancer metastases can occur de novo, in which metastatic foci are present at the moment of the initial diagnosis, which means that cancer has already spread prior to its detection. The other form of metastases presentation is the disease relapse in a form of metastatic disease definitive treatment.

Metastases from breast primary cancers have predominately been reported in the lymph nodes, lungs, bones, and liver in addition to the brain [7]. Central nervous system (CNS) metastasis from breast cancer is usually a late feature of metastatic disease [7]. Recent advances in systemic therapies result in an increased rate of long-term survivors, hence an increased incidence of CNS metastasis [8]. Symptomatic CNS metastasis usually appears after three to four months of spread in 5%-8% of breast cancer cases [8].

CNS metastasis can be either parenchymal or leptomeningeal metastasis. Leptomeningeal metastasis or carcinoma, also known as neoplastic meningitis or meningiosis or leukemic meningitis is the malignant infiltration of pia mater, arachnoid mater, and subarachnoid space. Leptomeningeal metastasis accounts for 2%-5% of patients with brain metastases from breast cancer [3].

In most cases, leptomeningeal metastasis occurs along with parenchymal metastasis, with only a few reported isolated cases of leptomeningeal metastasis in literature, thus actual incidence is unknown. It is diagnosed by lumbar puncture and cerebrospinal fluid (CSF) cytology, although the sensitivity is low, making a premortem diagnosis difficult and controversial. There are limited therapeutic options available making the prognosis abysmal. We present a case of isolated leptomeningeal metastasis occurring in a chemotherapy responsive breast cancer.

This article was previously presented as a poster at the BASO Conference in 2019 and the abstract is published in the European Journal of Surgical Oncology in 2019.

## Case presentation

**Figure 1:**
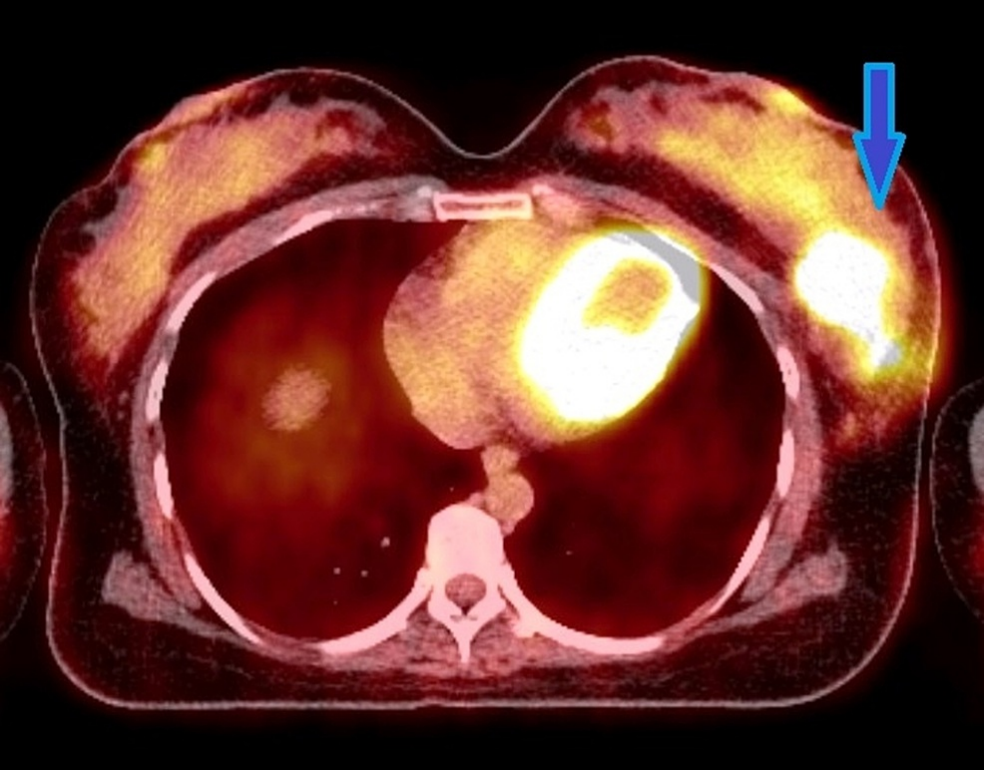
PET CT axial view showing an FDG avid soft tissue mass in the left breast.

A 37-year-old Asian female presented to the emergency department with sudden onset of multiple neurological symptoms including back pain, bilateral loss of sensation in lower limbs, dysphagia, dysarthria, and right ear tinnitus. She was treated for pulmonary tuberculosis 10 years ago and was diagnosed with a grade three invasive ductal carcinoma of the left breast eight months prior to this presentation. It was a unifocal tumor measuring 34 x 24 mm in size with multiple left axillary, left internal mammary, and left supraclavicular fossa lymph node involvement (Figure 1).

On immunohistochemistry, the tumor cells were triple-negative with a Ki-67 proliferative factor of 70%. She received six cycles of neoadjuvant chemotherapy with fluorouracil, epirubicin, and cyclophosphamide. The primary breast tumor was responsive to this regimen as evidenced by the reduction of size to 25 x 14 mm on a breast ultrasound conducted after receiving three months of chemotherapy. She was awaiting breast surgery for further management of cancer.

**Figure 2:**
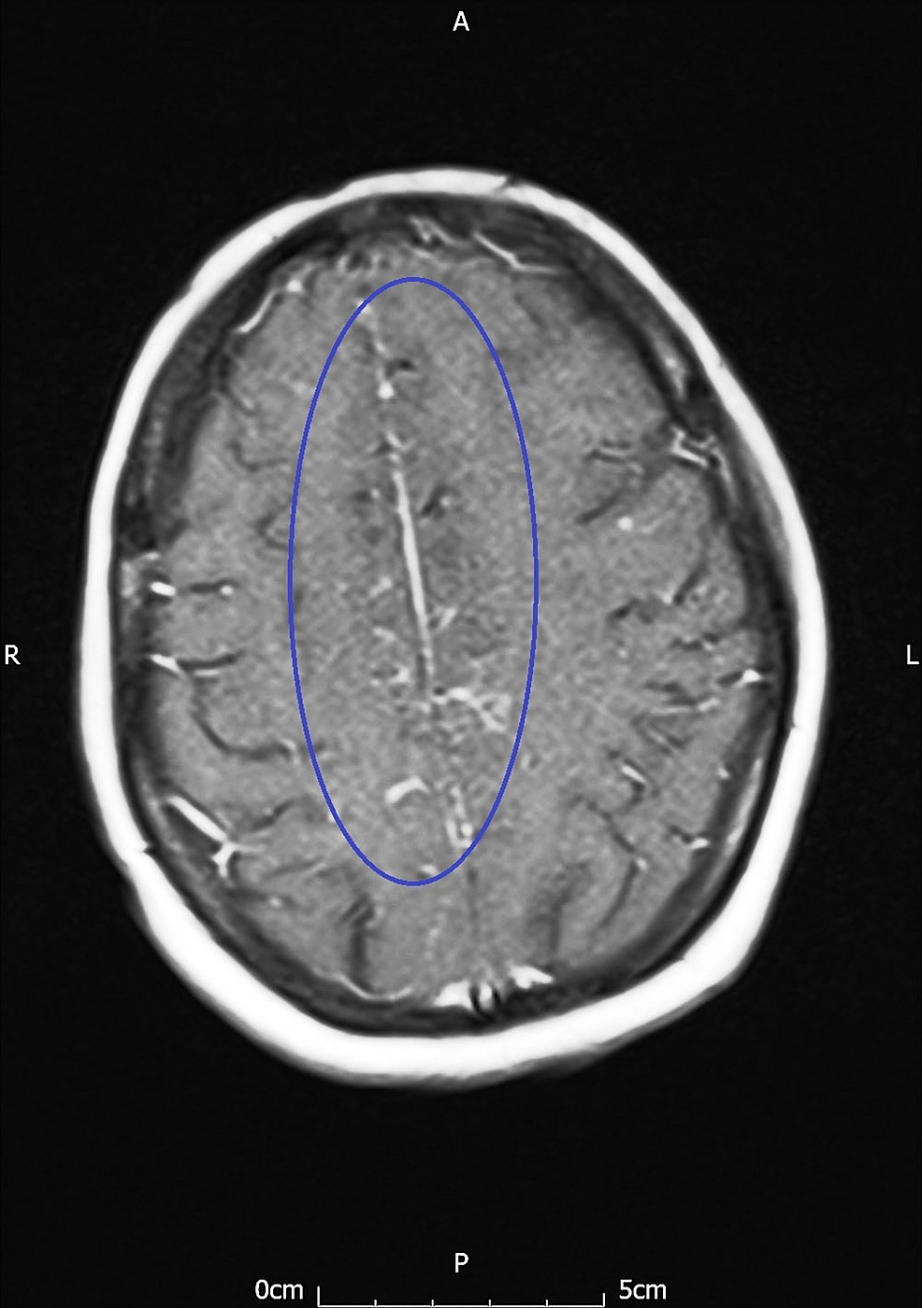
T1W axial post-contrast MRI head showing leptomeningeal enhancement in the parafalcine cerebral hemisphere.

**Figure 3:**
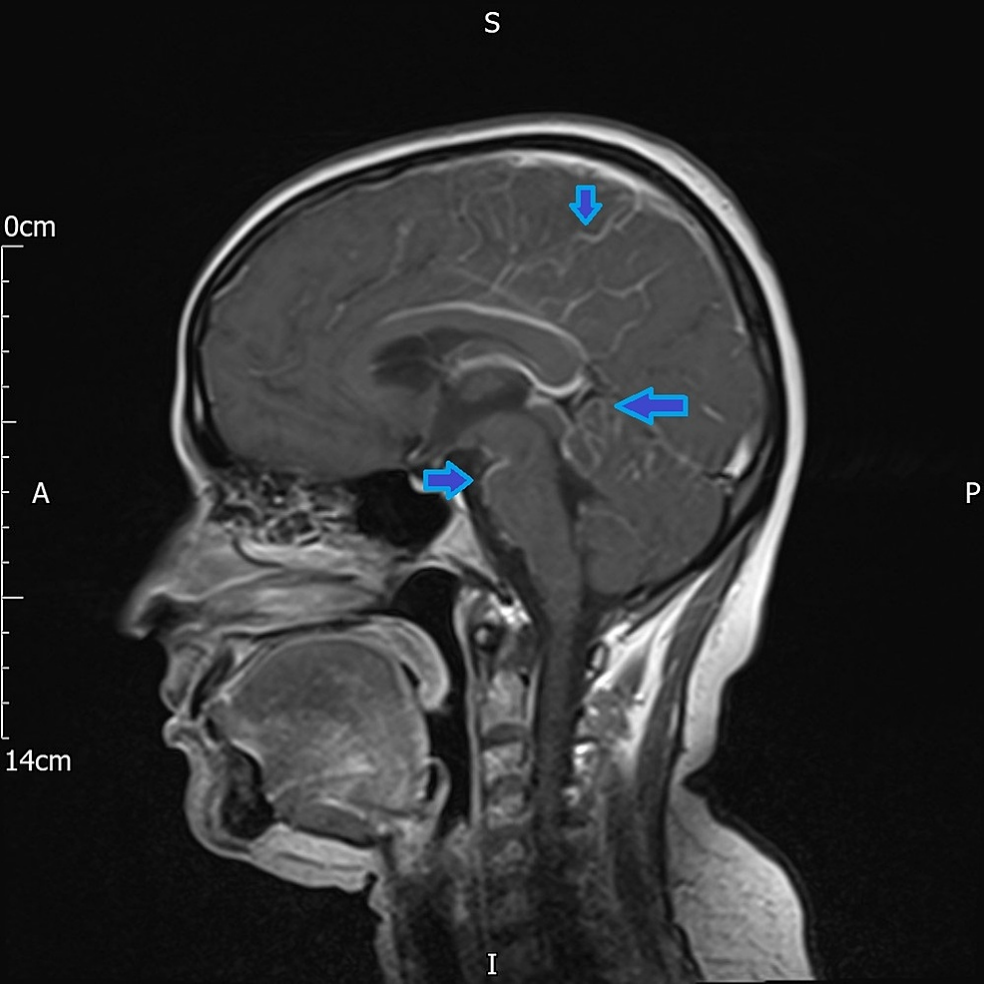
T1W Sagittal post-contrast MRI head showing leptomeningeal enhancement in the superior cerebellum.

She presented with sudden onset of rapidly progressive multiple neurological symptoms. On examination, there was reduced sensation and power in both lower limbs, multiple cranial nerve palsies, and bilateral left beat nystagmus. She also had altered cognition and reduced memory. There was no history of fever or rash, ruling out infective meningitis. Computed tomography (CT) scan of the head and gadolinium-enhanced magnetic resonance imaging (MRI) of the head and spine were conducted for further evaluation and diagnosis. The MRI scan revealed leptomeningeal enhancement and arachnoiditis in the superior cerebellum and the para-falcine cerebral hemisphere (Figures 2, 3).

**Figure 4:**
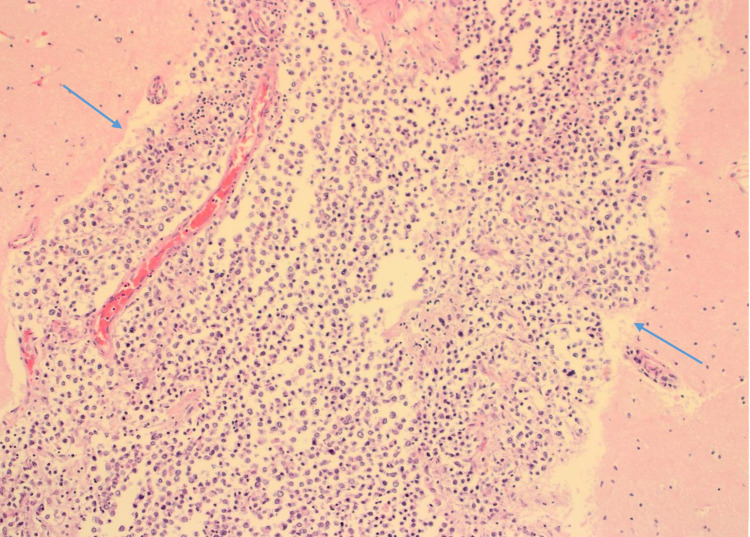
Brain H&E stain (x100) shows meninges extensively infiltrated by carcinoma cells. The cerebral tissue on either side of the meninges is uninvolved.

On CSF analysis, there was the presence of lymphocytosis with high protein and low glucose levels. The gram stain and acid-fast bacilli were negative. The CSF cytology was negative for malignant cells. Owing to the negative CSF cytology, the CSF fluid findings, and a past medical history of treated pulmonary tuberculosis, the differential diagnosis of tuberculous meningitis and CNS metastasis of the breast cancer was made. She was given supportive treatment and a trial of anti-tubercular medications. Although systemic chemotherapy was considered, the patient’s clinical condition deteriorated very rapidly and the patient succumbed due to worsening of the neurological symptoms. On autopsy, the histopathology analysis of the leptomeninges revealed a confirmed diagnosis of isolated leptomeningeal carcinomatosis (LC), where meninges were extensively infiltrated by carcinoma cells (Figure 4).

**Figure 5:**
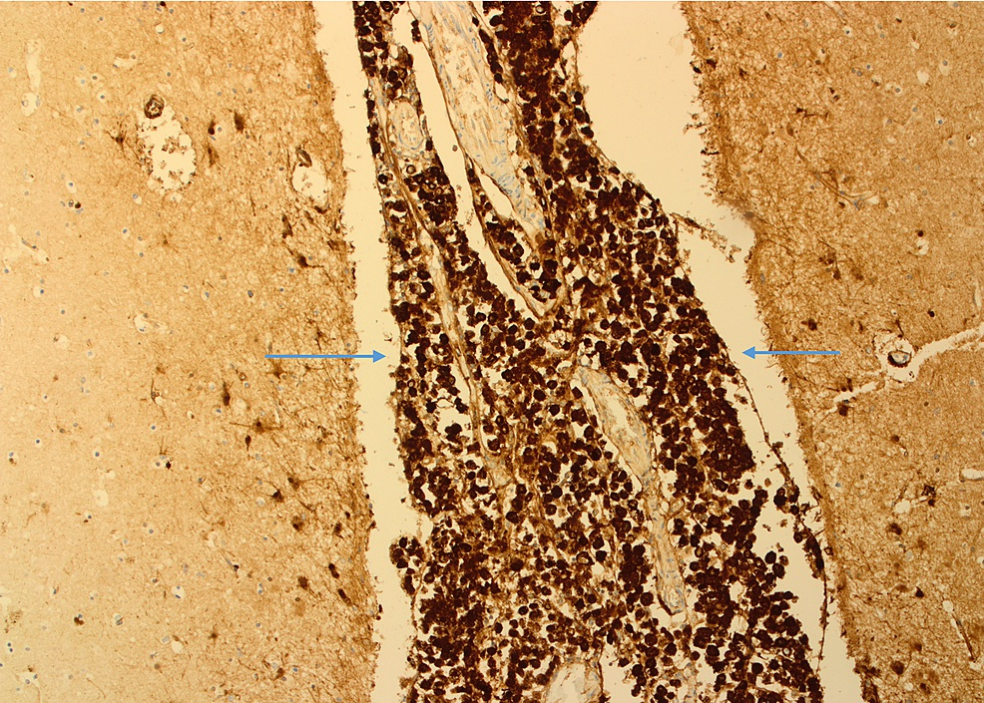
Keratin: Cam 5.2 stain (x40) shows positive carcinoma cells in meninges. Cerebral tissue on either side is uninvolved.

The immune-histochemistry staining showed that tumor cells are positive for Cam 5.2 (Figure 5).

**Figure 6:**
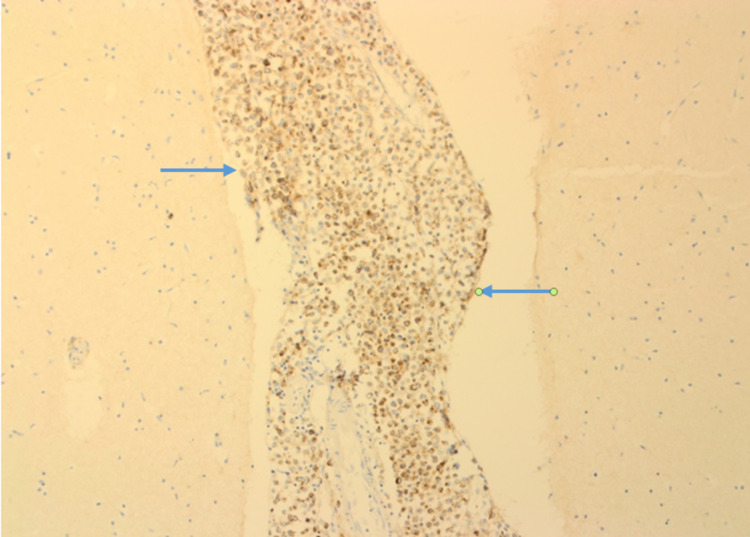
The invasive ductal carcinoma cells in meninges show positivity for the Echaderin stain. The cerebral tissue on either side is uninvolved.

The tumor cells are also positive for the Echaderin stain on the immunohistochemistry staining (Figure 6).

## Discussion

**Figure 7:**
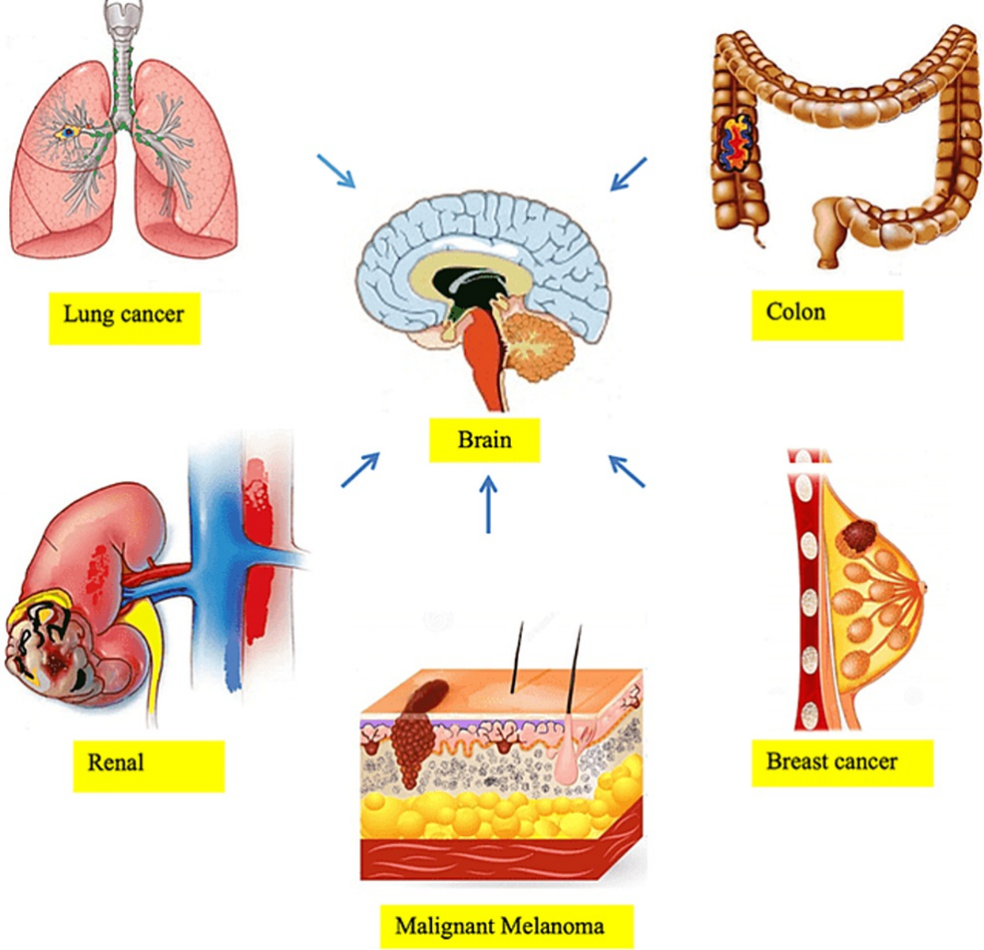
The common sites of primary extra-cranial malignant neoplasms that metastasize to the central nervous system. This illustration is created by the article author Abdalla Saad Abdalla Al-Zawi

LC is known to be caused by 1%-5% of solid malignancies, seen in 5%-15% of hematopoietic and lymphoid malignancies as well as detected in 1%-2% of primary brain tumor cases [9]. Brain metastasis from the extra-cranial origin is the most common cause of malignant neoplasm in the central nervous system, their rate is four times higher than that of the primary tumors[10]. Where astrocytoma or glioblastoma (which arises in brain glial cells) is recognized as the most common primary malignant brain tumor, breast cancer is reported to be the second most common source of CNS metastasis after lung cancer [11-13]. LC is most commonly caused by breast cancer, comprising 5% of metastatic breast cancer, malignant melanomas are other common solid tumors causing leptomeningeal metastasis in addition to non-small cell lung carcinomas (Figure 7) [8-14].

With only a few cases reported in the literature of isolated leptomeninges involvement, the incidence still remains unknown [15,16]. The overall incidence of leptomeningeal metastases is rising due to advances in chemotherapy regimens, improved diagnostic imaging techniques, and the reduced penetration of the blood-brain barrier by the currently available systemic agents [16]. Hormone-receptor-negative breast cancers are four times more likely to develop CNS metastasis [17].^ ^The tumor cells could spread through the hematogenous route, direct extension, bone-based metastasis, and/or perineural spaces. Isolated LC could be due to vertebral or paravertebral metastasis. Owing to the absence of immune surveillance in the CSF, the tumor cells remain protected in the leptomeninges, worsening the prognosis [18].

The clinical presentation of leptomeningeal metastasis involves an acute onset of rapidly progressive multifocal neurological symptoms, affecting the motor, sensory, and/or cognitive functions. Due to the spread of tumor cells to any area of the CNS via the CSF, the initial presentation of LM can be varied. The symptoms can be classified according to the affected neural structures; cerebral, spinal, and/or cranial nerves. These can range from headache, nausea, memory loss, motor weakness, and sensory loss up to seizures and encephalopathy.

High clinical suspicion is necessary for patients with breast cancer presenting with acute onset neurological symptoms. The diagnosis depends on high clinical suspicion, neuro-imaging, and CSF studies in patients with breast cancer presenting with acute onset neurological symptoms. The current imaging modality of choice is a gadolinium-enhanced, T1-weighted, multiplanar MRI scan of the head and spine, which is known to be more sensitive than a contrast CT scan. Leptomeningeal enhancement, nodular enhancement, and/or nervous enhancement are characteristic findings. Despite the low sensitivity, the gold standard for diagnosing leptomeningeal metastasis is the presence of malignant cells in CSF cytology. On the first lumbar puncture, the sensitivity of CSF cytology is only 50%-60%, rising up to 90% after the third sample[19]. Therefore, repeating the lumbar puncture is recommended where the first sample shows negative results; however, there are no known standardized interval gaps. Wasserstrom et al have recommended in a study of 90 patients that the diagnosis can be made purely on clinical evaluation in patients with a confirmed cancer diagnosis presenting with unexplained multifocal neurological dysfunction [19]. Non-specific CSF findings such as low glucose or raised proteins can be used as diagnostic aids in cases with high suspicion of leptomeningeal metastasis. Conversely, in patients with non-specific clinical features, more definitive parameters such as positive cytological reports, myelography showing tumor nodules, or CT scan revealing contrast-enhanced basal cisterns are useful [19].

Triple-negative breast cancer has a higher likelihood with a shorter interval of leptomeningeal disease development. There is no currently accepted standard approach for the management of this disease, although chemotherapy and radiotherapy play important roles. The survival time is usually very short, up to 16 weeks once LC has been confirmed [19].

Treatment decisions should be influenced by a thorough clinical assessment and risk stratification considering the patient’s functional status, primary tumor burden, and patient’s willingness for further treatment. A rapid decline in the clinical condition should be a guide to pursuing early palliative care. Currently, intrathecal methotrexate, systemic chemotherapy with or without localized external beam radiotherapy is the most commonly used treatment approach. Surgery is considered in cases of hydrocephalus. The her-2-neu positive disease has shown some response to intrathecal trastuzumab, lapatinib, and capecitabine. Despite the toxicity of intrathecal chemotherapy, many trials have compared various intrathecal chemotherapy regimens (methotrexate, thiotepa, cytarabine, and liposomal cytarabine) for LC with no significant differences in survival outcomes. The one-year survival rate is only 16%, making LC a rapidly fatal and devastating complication of breast cancer, despite the recent advances in the treatment modalities [20].

## Conclusions

To conclude, isolated LC is a rare fatal complication of breast cancer with a poor prognosis. The notable aspects of this case included the development of an isolated LC despite the good local response to the chemotherapy. High clinical suspicion is needed for an early diagnosis with neuro-imaging and CSF cytology. Systemic chemotherapy remains the current treatment modality. As the currently available diagnostic and therapeutic tools have limitations, further research is warranted to understand the pathophysiology of the isolated leptomeningeal metastasis to develop more innovative treatment options.
